# Anxiety, emotional abuse, and physical activity as correlates of AI-related affective dependence among adolescents: mediation, moderation, and network analyses

**DOI:** 10.3389/fpubh.2026.1956596

**Published:** 2026-10-06

**Authors:** Jingyu Yang, Jing Wang, Xiaodi Ma, Ning Wang, Ting Xiao, Ye Wu, Liying Lan, Yang Liu, Yanfei Wang

**Affiliations:** 1 Chengdu University, Chengdu, China; 2 Department of Anesthesiology for Surgery, Xilingol League Central Hospital, Xilin Hot, Inner Mongolia, China; 3 Heilongjiang University of Chinese Medicine, Harbin, China; 4 School of Physical Education, Chengdu Vocational University of the Arts, Chengdu, China; 5 Jishou University, Jishou, China; 6 Jilin Medical University, Jilin City, China; 7 Affiliated Hospital of Jilin Medical University, Jilin City, China; 8 Chengdu Experimental Middle School, Chengdu, China; 9 Department of Science and Education, Xilingol League Central Hospital, Xilinhot, Inner Mongolia, China

**Keywords:** adolescents, AI-related affective dependence, anxiety, emotional abuse, physical activity

## Abstract

**Objective:**

Drawing on attachment and emotion-regulation perspectives, this cross-sectional study examined the associations among emotional abuse (EA), anxiety, physical activity (PA), and AI-related affective dependence (hereafter, AI dependence) among adolescents.

**Methods:**

In November 2025, a total of 1,589 Chinese adolescents completed self-report questionnaires assessing EA, anxiety, PA, and AI dependence. Descriptive analyses, Pearson correlations, mediation and moderated mediation models, and a covariate-adjusted partial-correlation network were conducted. Common method bias was evaluated using both Harman’s single-factor test and a CFA-based unmeasured latent method construct (ULMC) approach.

**Results:**

EA was positively associated with anxiety and AI dependence and negatively associated with PA. Anxiety statistically accounted for a small but significant proportion of the EA-AI dependence association [indirect effect = 0.034, 95% CI (0.013, 0.055), 16.43% of the total association]. PA moderated the EA-anxiety association; the positive association was weaker among adolescents reporting higher PA. In the network analysis, the EA-anxiety edge was the strongest conditional association, and EA showed the highest node strength, although these findings should be interpreted descriptively rather than causally.

**Conclusion:**

The findings suggest that anxiety and PA are meaningful correlates in the association between EA and AI dependence. Because the data are cross-sectional, the results indicate statistical associations rather than temporal or causal mechanisms.

## 1 Background

Emotional abuse (EA) is a nonphysical form of child maltreatment characterized by harmful caregiver–child interactions, including insults, humiliation, threats, rejection, and the withholding of emotional support (1). In the present study, EA was operationalized broadly to capture emotional maltreatment experiences involving both emotionally abusive and neglect-related features. It typically involves enduring, harmful caregiver–child interactions (2). Its consequences may be less visible than physical injury, yet they can persist into adulthood and undermine self-esteem, emotional security, mental health, social functioning, and intimate relationships (3, 4). Accordingly, research commonly assesses the abuse and neglect components with the Childhood Trauma Questionnaire (CTQ) (5, 6). Meta-analytic estimates place the prevalence of EA at 36.3% worldwide (7) and 36.7% among Chinese adolescents (8). Among young people, EA has also been associated with post-traumatic stress disorder (9), depressive symptoms (10), altered immune functioning (11), and lower self-esteem (12). Clarifying the processes through which EA affects adolescents is therefore important for targeted mental health prevention.

Generative artificial intelligence (AI), including ChatGPT, Kimi, Siri, Copilot, and Doubao, has become embedded in adolescents’ academic, creative, social, and emotional routines. Because cognitive and emotional regulatory systems are still developing during adolescence, frequent interaction with highly responsive AI agents may be associated with a form of psychological reliance that differs from conventional internet or smartphone overuse (13, 14). In the present study, AI-related affective dependence (hereafter, AI dependence) refers specifically to adolescents’ psychological and emotional reliance during human-AI interaction. This construct is not equivalent to AI addiction or problematic AI use, which usually involve additional features such as impaired control, functional impairment, tolerance, or withdrawal-like experiences (14, 15).

Anxiety is a response to uncertainty or perceived danger that can be adaptive at modest levels but disruptive when persistent or excessive (16, 17). Adolescents may be especially sensitive because they are simultaneously negotiating identity and social change. Meta-analyses estimate that anxiety affects approximately 20–25% of adolescents worldwide (18, 19). Attachment and emotion regulation perspectives suggest that insecure early relationships and maladaptive regulatory strategies can predispose individuals to anxiety (20, 21). Neglect, humiliation, and rejection may erode felt security and promote hypervigilance and negative self-appraisal (22). Adolescents experiencing anxiety may then turn to digital media or intelligent systems for relief (23, 24). Although responsive, nonjudgmental AI can temporarily soothe distress, repeated use for this purpose may strengthen psychological reliance. We therefore hypothesize that anxiety mediates the association between EA and AI dependence (H2).

Physical activity (PA) includes both planned exercise and routine bodily movement that increases energy expenditure (25). Beyond its physical benefits, PA contributes to stress recovery and emotional regulation (26, 27). Regular activity may alleviate anxiety by lowering physiological arousal and influencing dopamine and serotonin pathways (28). It may be especially valuable for adolescents exposed to EA because exercise can strengthen self-efficacy, social contact, and emotional resilience (29). Evidence that PA reduces the emotional burden associated with childhood maltreatment further supports this possibility (30). We thus hypothesize that PA moderates the EA–anxiety association, with a weaker positive association at higher levels of PA (H3).

Taken together, the proposed model treats EA as a correlate of AI dependence, anxiety as a statistical mediator, and PA as a moderator of the EA-anxiety association. The ordering of EA before anxiety was selected because developmental and attachment-based accounts generally regard early emotional maltreatment as a distal vulnerability factor for later internalizing symptoms; nevertheless, cross-sectional data cannot exclude reverse or bidirectional processes, such as anxiety shaping the recall or salience of adverse experiences. We therefore tested the following hypotheses using association-based language: H1, EA would be positively associated with AI dependence; H2, anxiety would statistically mediate the association between EA and AI dependence; and H3, PA would moderate the association between EA and anxiety, such that this association would be weaker at higher levels of PA. The network analysis was exploratory and was used to describe the conditional association pattern among the focal variables after demographic adjustment (See Figure 1).

**Figure 1:**
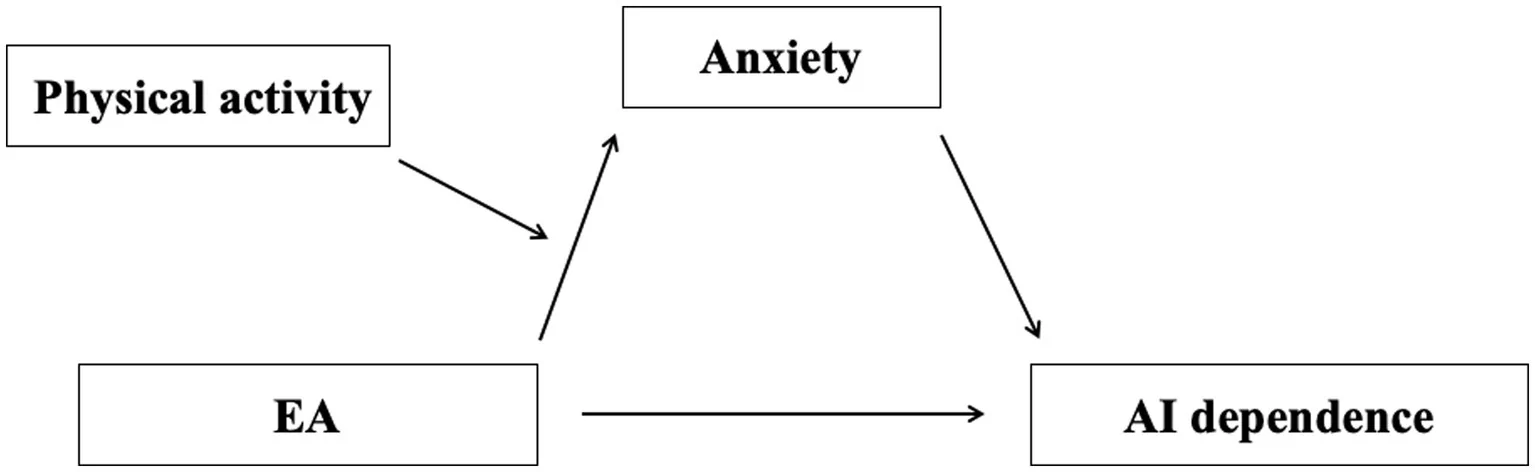
Model assumption diagram. AI dependence refers to AI-related affective dependence; the same abbreviation is used consistently in all figures.

## 2 Methods

### 2.1 Participants

Data were collected in November 2025 from 1,705 students enrolled in six primary, middle, and high schools in Hunan, Sichuan, Shaanxi, Shanxi, and Anhui provinces, China. Ethical approval was granted by the affiliated institution’s Institutional Review Board in November, 2025, before participant recruitment and data collection. Written informed consent was obtained from students’ legal guardians, and adolescent assent/consent was obtained before participation. With assistance from homeroom teachers, students completed paper questionnaires in their classrooms. They were informed of the study purpose, intended use of the data, confidentiality protections, voluntary nature of participation, and their right to withdraw at any time. Responses were anonymous, and questionnaire completion required approximately 10 min. Of the 1,705 respondents, 1,589 had eligible data for all focal variables and covariates required for the primary analyses and therefore constituted the main analytical sample (93.2%). The sample included 765 boys (48.1%) and 824 girls (51.9%), with a mean age of 14.62 ± 1.65 years. There were 225 elementary school students (14.2%), 933 junior high school students (58.7%), and 431 senior high school students (27.1%). A total of 369 participants (23.2%) were only children, whereas 1,220 (76.8%) had siblings. Rural and urban household registration accounted for 136 (8.6%) and 1,453 (91.4%) participants, respectively. Maternal education was primary school or below for 292 participants (18.4%), secondary school for 739 (46.5%), university for 221 (13.9%), graduate level for 25 (1.6%), and unknown for 312 (19.6%). The corresponding paternal education figures were 237 (14.9%), 769 (48.4%), 255 (16.0%), 34 (2.1%), and 294 (18.5%), respectively. Family structure included 1,017 nuclear families (64.0%), 294 three-generation households (18.5%), 163 single-parent families (10.3%), 52 grandparent-led households (3.3%), and 63 other family arrangements (4.0%). A total of 470 adolescents (29.6%) were classified as left-behind children, whereas 1,119 (70.4%) were not. On the 1–10 self-rated family economic status scale, scores of 5 (*n* = 611, 38.5%) and 6 (*n* = 305, 19.2%) were the most frequently reported, indicating that most participants perceived their family economic circumstances as moderate to moderately favorable.

### 2.2 Measures

#### 2.2.1 Emotional abuse

EA was operationalized with the psychological abuse and psychological neglect subscales from the revised Childhood Trauma Questionnaire–Short Form (CTQ-SF) (31). Two of the four items assess abuse and two assess neglect; neglect items were reverse-scored. Each item uses a 5-point response format from 1 (never) to 5 (always), producing a total between 4 and 20, with larger values denoting greater EA. Cronbach’s α was 0.755 for psychological abuse, 0.737 for psychological neglect, and 0.788 for the combined measure. Previous work supports the reliability and validity of the CTQ-SF (32). Because the abbreviated measure combines psychological abuse and psychological neglect items, EA in this manuscript should be understood as emotional abuse-related emotional maltreatment rather than an abuse-only construct.

#### 2.2.2 Anxiety

Anxiety was measured with the two-item Generalized Anxiety Disorder Scale (GAD-2) (33). Items were scored from 0 (not at all) to 3 (nearly every day), yielding total scores from 0 to 6, consistent with the commonly validated GAD-2 scoring procedure; larger scores indicate more severe anxiety. Internal consistency in the present sample was good (Cronbach’s *α* = 0.862).

#### 2.2.3 AI-related affective dependence scale

AI-related affective dependence was assessed using an adapted version of the craving dimension of the Emotional Dependence Scale (34), which was originally developed to assess affective dependence in close interpersonal relationships. The adaptation was intended to preserve the core psychological meaning of affective dependence while modifying the target, wording, and situational context for human-AI interaction. References to romantic partners or interpersonal relationship targets were replaced with references to AI or interactions with AI, and items retaining a strong interpersonal or romantic connotation were further revised to better reflect adolescents’ psychological concern, emotional needs, and reliance on generative AI. The adaptation was also informed by emerging research on adolescent relationships with AI and on AI dependence or problematic AI use (13–15). Content validity was evaluated by five experts in psychology, adolescent health, and digital behavior research. The experts assessed the adapted items for construct relevance, semantic appropriateness, clarity, and comprehensibility for adolescents. Several expressions were refined based on their feedback before the formal survey. The item-level content validity index (I-CVI) ranged from 0.80 to 1.00, and the scale-level average content validity index (S-CVI/Ave) was 0.96, indicating good content validity. Before the formal survey, a pretest and cognitive feedback procedure was conducted with adolescents to assess whether the items were understandable, naturally worded, and consistent with their experiences of generative AI use. Minor wording revisions were made accordingly. The adapted measure was therefore intended to capture AI-related affective dependence, defined here as psychological and emotional reliance on AI during human-AI interaction, rather than clinical AI addiction, problematic AI use, or general AI-use frequency (14, 15). In the formal analytical sample, the five-item scale demonstrated excellent internal consistency (Cronbach’s *α* = 0.946). Exploratory and confirmatory factor analyses supported a one-factor structure. The KMO value was 0.892, Bartlett’s test was significant (*p* < 0.001), all factor loadings exceeded 0.70, composite reliability was 0.890, and the average variance extracted was 0.620. These findings provided preliminary support for the internal consistency and factorial structure of the adapted measure in the present adolescent sample.

To further examine discriminant validity, we conducted a supplementary analysis using data from the same school-based survey in which AI-related affective dependence and short-form video addiction were assessed concurrently. A total of 1,676 participants had available data for both measures and were included in this analysis. This number exceeded the main analytical sample of 1,589 because the supplementary discriminant-validity analysis involved only these two constructs, whereas the primary analyses required eligible data across all focal variables and covariates. Thus, the difference in sample size reflected the different data requirements of the two analyses rather than the use of a separate sample. The supplementary analysis was covered by the same institutional ethical approval and consent/assent procedures described above. Short-form video addiction was selected as an external comparison construct because both measures reflect dependence-like experiences in digital environments, while differing in their target and psychological function. The correlation between the two total scores was modest (*r* = 0.219, *p* < 0.001), suggesting that the constructs were related but not redundant. A two-factor CFA specifying AI-related affective dependence and short-form video addiction as separate latent constructs showed substantially better fit than a one-factor model in which all 13 items loaded onto a single digital-dependence factor. The two-factor model yielded CFI = 0.919, TLI = 0.901, RMSEA = 0.085, 90% CI [0.081, 0.089], and SRMR = 0.045, whereas the one-factor model showed poor fit [CFI = 0.473, TLI = 0.368, RMSEA = 0.216, 90% CI (0.212, 0.220), SRMR = 0.276]. The differences in fit indices were substantial (ΔCFI = 0.446; ΔRMSEA = −0.131). The HTMT value was 0.237, well below the commonly used 0.85 criterion. Together, these findings provide supplementary evidence that AI-related affective dependence is empirically distinguishable from short-form video addiction. Nevertheless, this comparison does not establish discriminant validity against other related constructs, such as general AI-use frequency, problematic AI use, problematic internet use, smartphone dependence, or functional impairment.

#### 2.2.4 Physical activity

PA was quantified with Liang (35) Physical Activity Rating Scale (PARS-3). Respondents rate exercise intensity, duration, and frequency on three 5-point items. The composite is calculated as Intensity × (Duration − 1) × Frequency and ranges from 0 to 100, with higher scores indicating more PA. Cronbach’s α was 0.709 in this sample. The brief format of the PARS-3 has made it suitable for large adolescent surveys (36).

### 2.3 Covariates

Analyses adjusted for gender, age, grade, only-child status, rural or urban residence, maternal and paternal education, family structure, left-behind status, and self-rated family economic status (1 = lowest, 10 = highest). These covariates were selected because adolescent technology use and emotional health may vary by demographic and family socioeconomic context. Variables such as academic stress, daily AI or screen-use duration, sleep duration, parental monitoring, loneliness, attachment style, emotion regulation, and motives for AI use were not available and are therefore considered in the limitations.

### 2.4 Statistical analysis

Analyses were performed in SPSS 27.0, Hayes’ PROCESS macro, R 4.2.1, and lavaan. Pearson correlations were first calculated. Common method bias was initially screened using Harman’s single-factor procedure and was further evaluated using a CFA-based unmeasured latent method construct (ULMC) model. The trait-only CFA specified four latent constructs (EA, anxiety, AI dependence, and PA), whereas the ULMC model additionally specified an orthogonal latent method factor with equal loadings on all observed indicators. Before the mediation and moderated mediation analyses, all focal continuous variables (EA, anxiety, PA, and AI-related affective dependence) were standardized as z-scores. Categorical covariates retained their original coding. Accordingly, coefficients reported for the focal variables are presented as standardized coefficients (β). Mediation was tested using PROCESS Model 4, and moderated mediation was tested using PROCESS Model 7, with 5,000 bootstrap samples and 95% confidence intervals. Both models were adjusted for gender, age, grade, only-child status, rural or urban residence, maternal and paternal education, family structure, left-behind status, and self-rated family economic status. Given the cross-sectional design, indirect effects were interpreted as statistical associations rather than evidence of temporal or causal mechanisms. For the network analysis, each focal total score was residualized for the same set of covariates before network estimation to characterize associations among EA, anxiety, PA, and AI dependence beyond demographic and family background. A Gaussian partial-correlation network was then estimated using the residualized variables. Because the network consisted of only four aggregate variables, regularization was not applied. Instead, edge accuracy was evaluated using 1,000 nonparametric bootstrap samples, and node strength was calculated as the sum of the absolute edge weights connected to each node. Given the small number of aggregate variables and the cross-sectional design, network centrality indices were interpreted descriptively and were not regarded as evidence of causal priority or intervention importance.

## 3 Results

### 3.1 Common method Bias test

Harman’s single-factor procedure extracted six unrotated factors with eigenvalues above 1. The first factor explained 15.944% of the total variance, below the conventional 40% threshold. We further conducted a CFA-based ULMC analysis. The trait-only CFA showed acceptable fit, chi-square = 440.020, df = 71, CFI = 0.956, TLI = 0.944, RMSEA = 0.057, 90% CI [0.053, 0.062], and SRMR = 0.033. After adding the latent method factor, model fit changed only slightly, chi-square = 371.226, df = 70, CFI = 0.964, TLI = 0.954, RMSEA = 0.052, 90% CI [0.048, 0.057], and SRMR = 0.031; delta CFI = 0.008 and the average standardized method variance was approximately 3.0%. These findings suggest that common method variance was unlikely to dominate the observed associations, although it cannot be fully ruled out.

### 3.2 Correlation analysis

EA correlated positively with AI dependence (*r* = 0.214, *p* < 0.001) and anxiety (*r* = 0.387, *p* < 0.001) and inversely with PA (*r* = −0.136, *p* < 0.001). Anxiety was also positively correlated with AI dependence (*r* = 0.166, *p* < 0.001) and inversely correlated with PA (*r* = −0.149, *p* < 0.001). PA showed a very small negative correlation with AI dependence (*r* = −0.044, *p* > 0.05), suggesting that PA was more closely related to EA and anxiety than to AI dependence at the bivariate level (See Figure 2).

**Figure 2:**
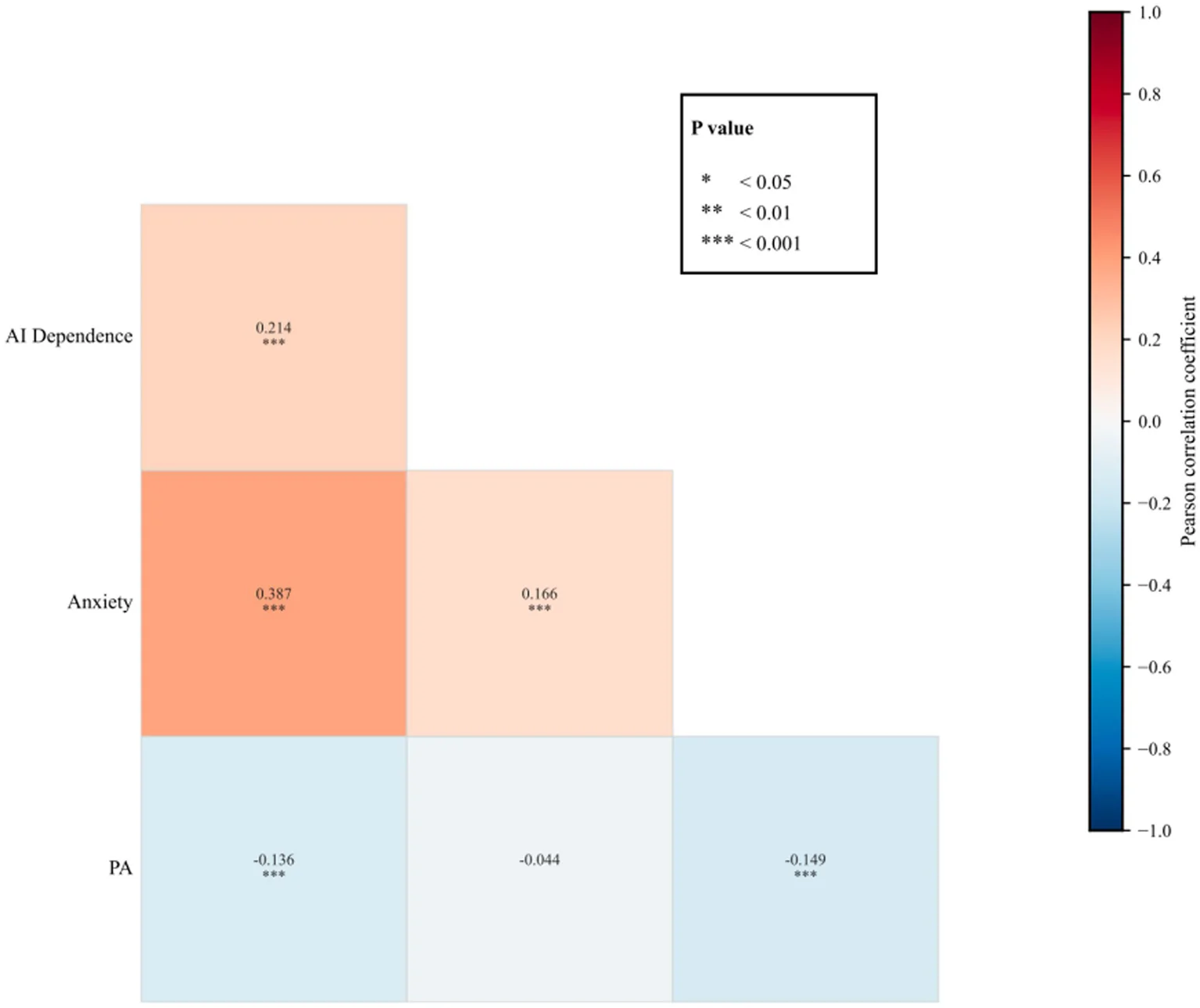
Correlation Heatmap.

### 3.3 Network analysis

After demographic adjustment, the largest conditional association in the network connected EA and anxiety [partial *r* = 0.340, bootstrap 95% CI (0.289, 0.394)]. EA was also positively connected with AI dependence [partial *r* = 0.159, 95% CI (0.104, 0.209)] and negatively connected with PA [partial *r* = −0.071, 95% CI (−0.122, −0.019)]. Anxiety retained a smaller positive edge with AI dependence [partial *r* = 0.087, 95% CI (0.035, 0.142)], whereas the PA-AI dependence edge was near zero [partial *r* = −0.010, 95% CI (−0.058, 0.041)]. Node strength was highest for EA [0.570, 95% CI (0.481, 0.653)], followed by anxiety [0.471, 95% CI (0.392, 0.555)], AI dependence [0.256, 95% CI (0.200, 0.335)], and PA [0.125, 95% CI (0.083, 0.195)]. This analysis complements the regression models by showing the unique conditional association pattern among all focal variables, but it does not identify causal pathways or intervention targets (See Figure 3).

**Figure 3:**
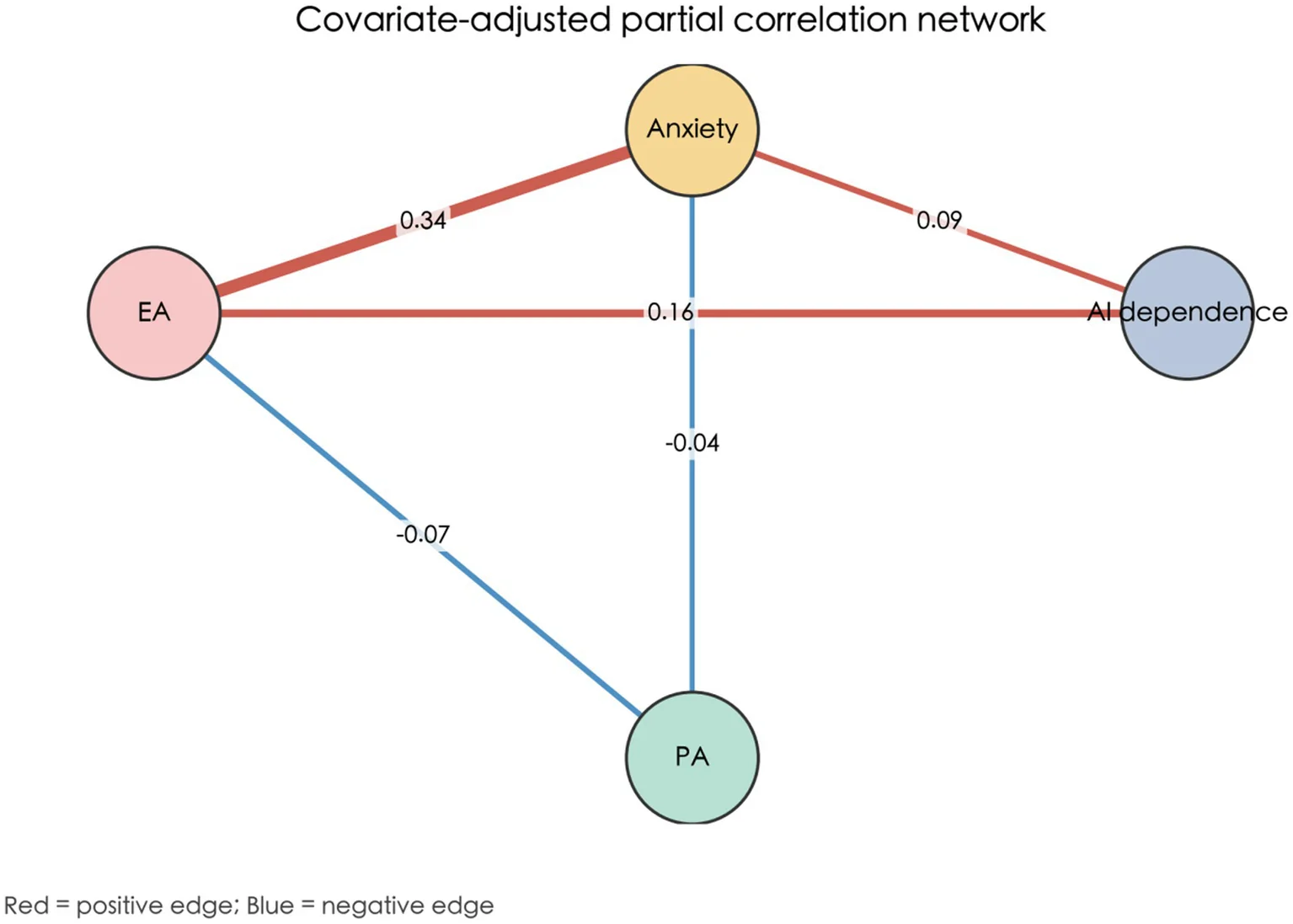
Covariate-adjusted partial correlation network.

### 3.4 Mediation path analysis

Before anxiety entered the model, EA was positively associated with AI dependence (*β* = 0.207, *p* < 0.001). Adding anxiety reduced the direct coefficient to *β* = 0.173 (*p* < 0.001), a difference of 0.034 representing 16.43% of the total association. The bootstrap confidence interval for the indirect association did not include zero [95% CI (0.013, 0.055)], indicating a statistically significant but small indirect association through anxiety. Thus, the mediation result should be understood as evidence that anxiety is a modest statistical link between EA and AI dependence, rather than proof of a causal process (See Tables 1, 2, and Figure 4).

**Table 1:** Mediation model test.

Outcome variable	Predictor variable	*β*	SE	*t*	*R* ^2^	*F*
AI Dependence	EA	0.207	0.025	8.238***	0.054	8.112***
Anxiety	EA	0.360	0.023	15.535***	0.194	34.472***
AI Dependence	EA	0.173	0.027	6.428***	0.061	8.504***
	Anxiety	0.095	0.027	3.490***		

*p<0.05; ***p<0.001.

**Table 2:** Mediation model path analysis.

Intermediate path	Effect size	SE	Bootstrap 95% CI	Proportion of mediating effect
Total effect	0.207	0.025	0.157, 0.256	
Direct effect	0.173	0.027	0.120, 0.225	
Total indirect effect	0.034	0.011	0.013, 0.055	16.43%

**Figure 4:**
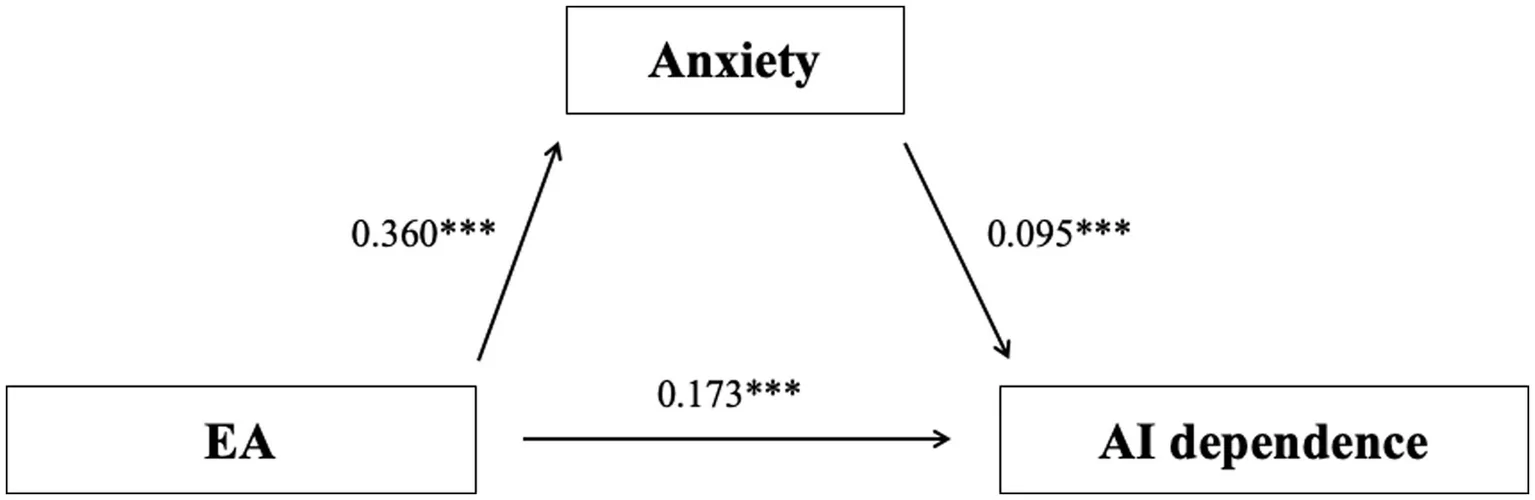
Mediation model diagram.

### 3.5 Moderation effect analysis

The moderated mediation model tested whether PA altered the EA-anxiety association (Table 3). EA remained positively associated with AI dependence (*β* = 0.173, *p* < 0.001) and anxiety (*β* = 0.356, *p* < 0.001), and anxiety was positively associated with AI dependence (*β* = 0.095, *p* < 0.001). The EA × PA interaction was significant (*β* = −0.086, SE = 0.023, *t* = −3.740, *p* < 0.001), indicating that the positive EA-anxiety association was weaker among adolescents with higher PA. The index of moderated mediation was −0.008, with a 95% bootstrap confidence interval of [−0.015, −0.002]. Because the design was observational and cross-sectional, this moderation should be interpreted as PA being a potentially modifiable correlate or protective condition, not as evidence that increasing PA would directly reduce anxiety or AI dependence (See Figures 5–7).

**Table 3:** Testing the moderated mediation model.

Outcome variable	Predictor variable	*β*	SE	*t*	*R* ^2^	*F*
AI Dependence	EA	0.173	0.027	6.428***	0.061	8.504***
	Anxiety	0.095	0.027	3.490***		
Anxiety	EA	0.356	0.023	15.384***	0.203	30.779***
	PA	−0.061	0.024	−2.499*		
	EA × PA	−0.086	0.023	−3.740***		

*p<0.05; ***p<0.001. The EA × PA interaction was *β* = −0.086, SE = 0.023, *t* = −3.740, *p* < 0.001. The index of moderated mediation was −0.008, 95% bootstrap CI [−0.015, −0.002].

**Figure 5:**
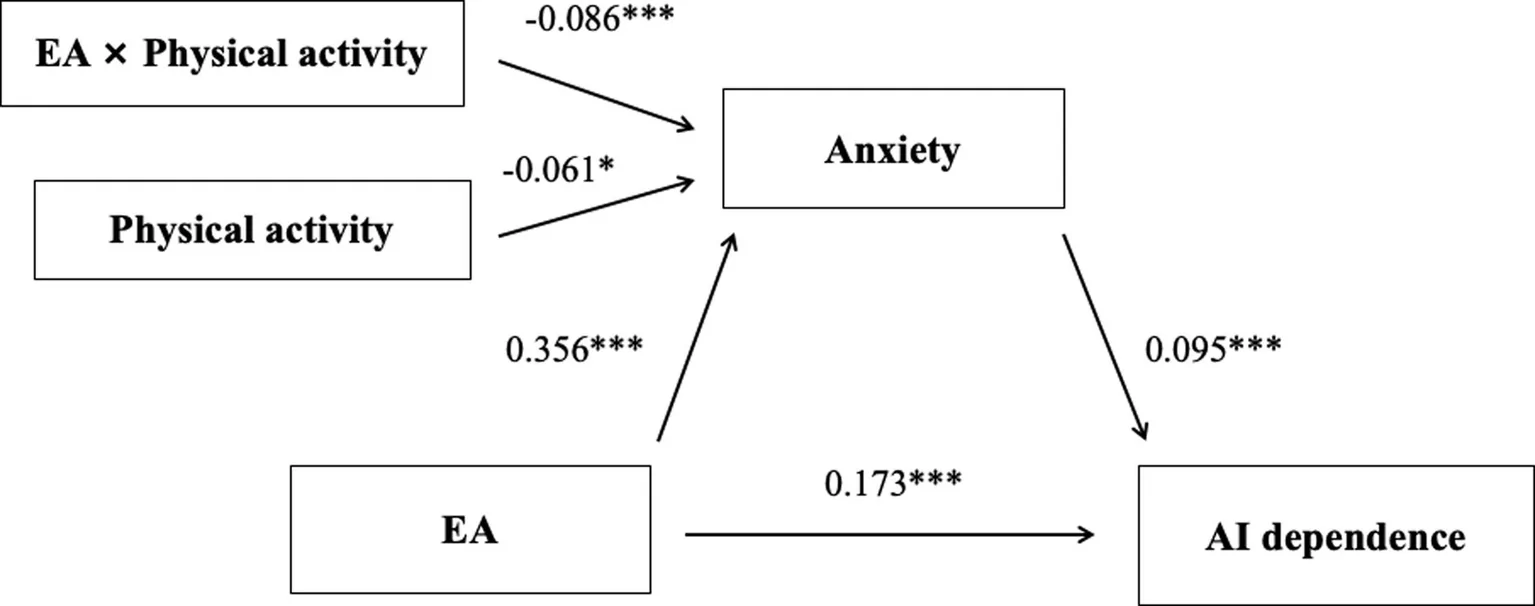
Moderated mediation model diagram.

**Figure 6:**
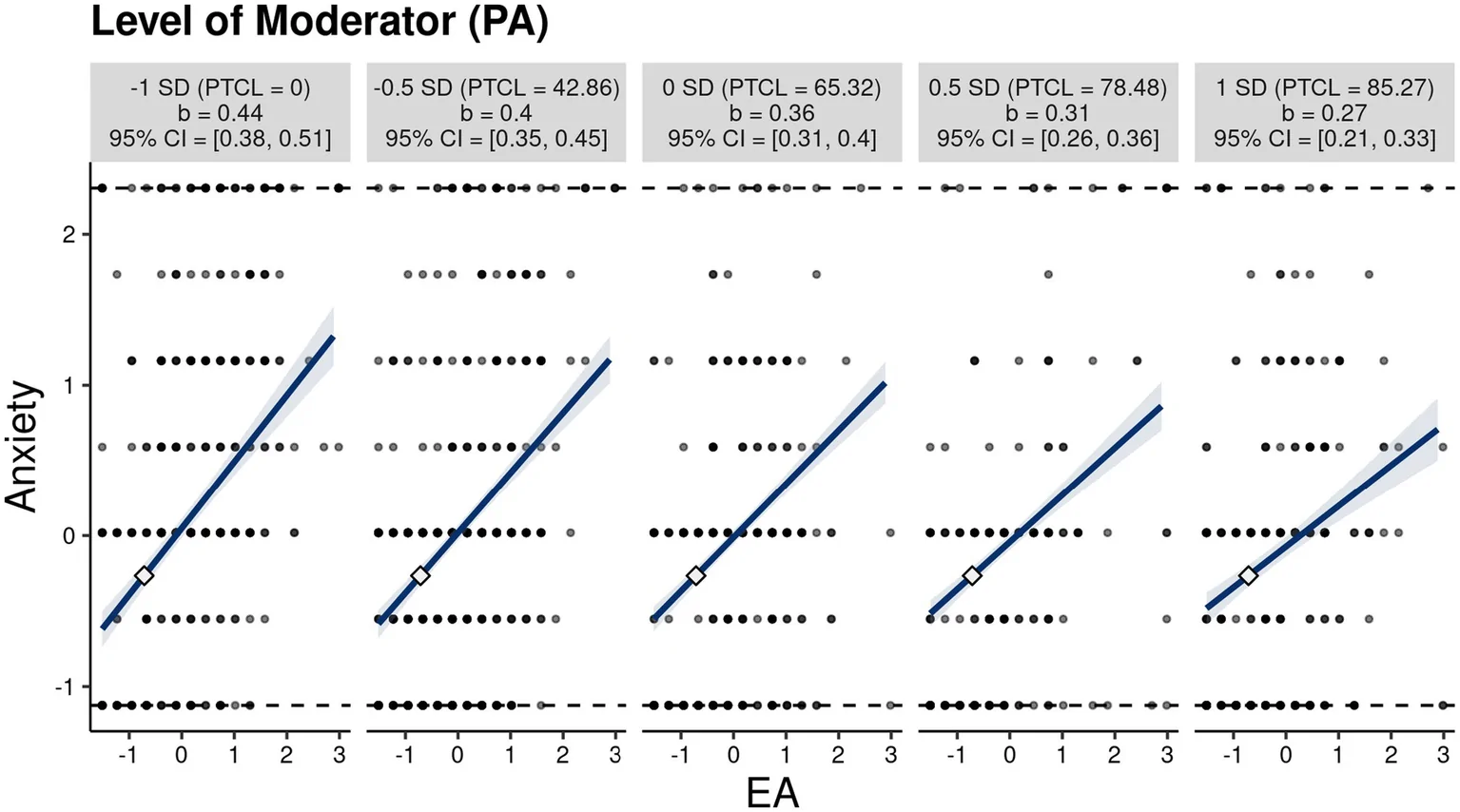
Simple slope plots of the focal predictor at different levels of PA as a moderator. The shaded areas represent the 95% confidence intervals (CIs), and the gray circles indicate the observed data points. All variables involved in the interaction analysis were standardized (z-scores). PTCL denotes percentile values.

**Figure 7:**
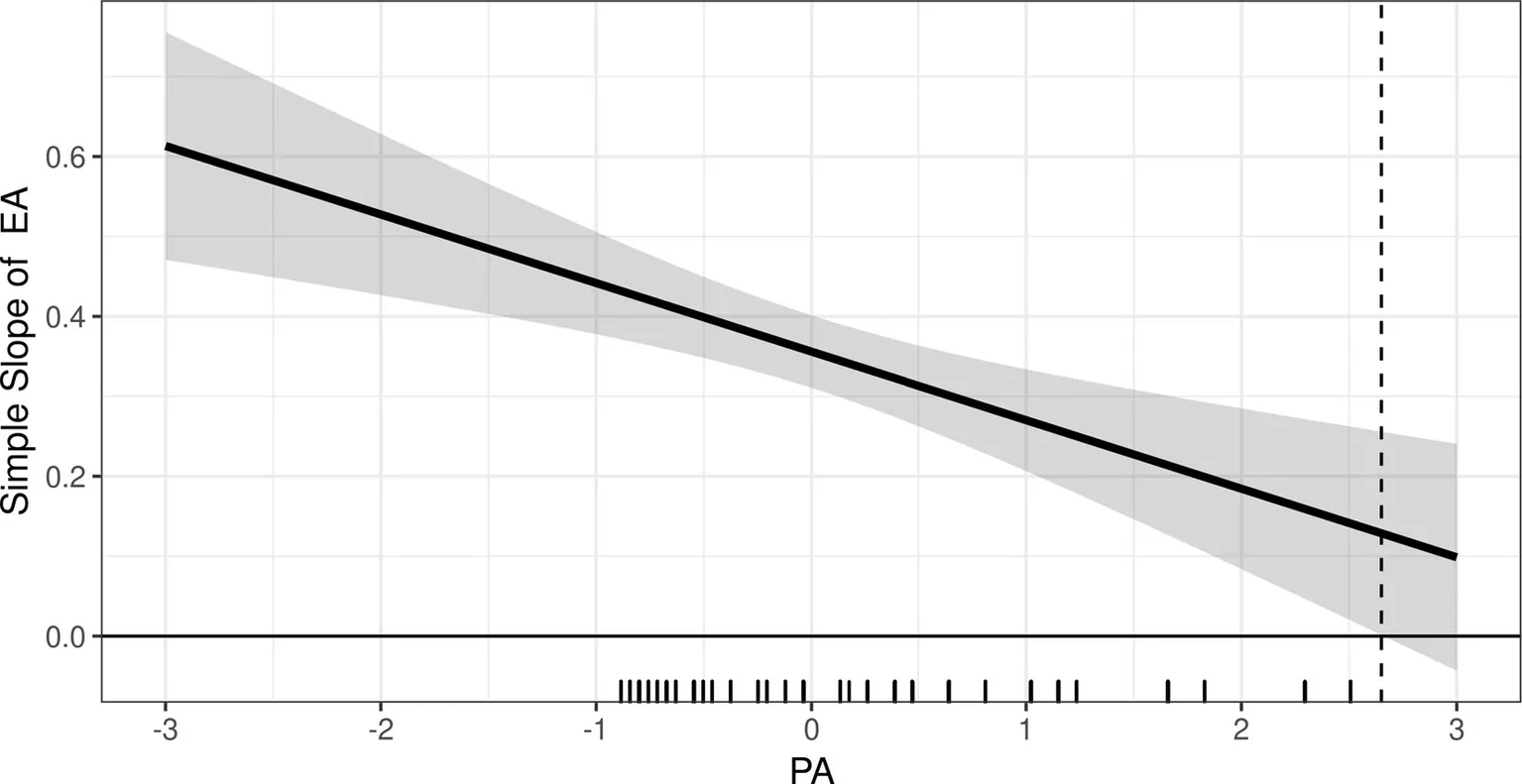
Johnson–Neyman (J–N) plot of the moderating effect of PA. The shaded areas represent the 95% confidence intervals (CIs).

## 4 Discussion

This study used attachment and emotion-regulation perspectives to examine how EA, anxiety, PA, and AI dependence were interrelated in a cross-sectional sample of adolescents. EA was associated with greater anxiety and stronger AI dependence and with lower PA; anxiety accounted for a small but significant portion of the EA-AI dependence association; and PA attenuated the EA-anxiety association. The network analysis provided a complementary descriptive view by showing that the EA-anxiety edge was the strongest conditional association after covariate adjustment, whereas PA had little direct conditional association with AI dependence.

The positive association between EA and AI dependence is consistent with the possibility that adolescents exposed to emotionally invalidating or neglectful environments may be more likely to seek predictable, nonjudgmental, and immediately responsive forms of digital interaction. From an attachment perspective, conversational AI may be experienced by some adolescents as a low-risk source of reassurance or companionship (13). However, this interpretation remains theoretical because attachment style, loneliness, social support, emotion-regulation strategies, and motives for using AI as emotional support were not directly measured. The present findings therefore support an association-based interpretation rather than a direct test of a compensatory attachment mechanism.

Anxiety statistically accounted for part of the association between EA and AI dependence. This pattern is consistent with studies linking childhood emotional maltreatment with internalizing symptoms and with evidence that adolescents experiencing anxiety may rely more heavily on controllable digital environments for reassurance (37, 38). At the same time, the indirect association was small in magnitude. Its practical meaning is therefore likely cumulative and context-dependent: anxiety may be one psychological correlate among several, rather than the dominant pathway through which EA is related to AI dependence. Reverse or reciprocal pathways are also plausible, because persistent reliance on AI for reassurance may reinforce avoidance of real-life interpersonal coping and thereby be associated with higher anxiety over time.

PA moderated the association between EA and anxiety, with a weaker positive EA-anxiety association among adolescents reporting higher PA. This result is consistent with literature suggesting that PA is related to stress regulation, mood improvement, and more adaptive coping (39). A plausible explanation is that PA provides physiological recovery, structured routines, peer interaction, and mastery experiences that may buffer the emotional correlates of adverse experiences (40). Importantly, PA was not directly associated with AI dependence in the adjusted network. One explanation is that AI use among students may often be instrumental or academic, whereas PA may be more strongly related to leisure-based screen behaviors such as gaming or social networking. Thus, PA should be framed as a potentially protective correlate in the EA-anxiety link rather than as a direct solution for AI dependence.

The study contributes theoretically by extending attachment and emotion-regulation accounts of EA to adolescents’ affective reliance on generative AI. Rather than equating AI dependence with traditional behavioral addiction, the revised interpretation emphasizes emotional reliance, reassurance seeking, and the possibility that AI may serve compensatory functions for some adolescents. Practically, the findings suggest several candidate areas for future intervention research, including anxiety management, supportive family and school climates, and opportunities for PA. These implications should remain cautious: the present study identifies correlates and statistical moderation, but it did not test whether any intervention can reduce AI dependence.

Several features distinguish this work. It brings AI dependence into research on adolescent emotional development and combines mediation, moderation, and network approaches. The mediation and moderation models quantify specific conditional indirect associations, whereas the network analysis summarizes the pattern of unique associations among the focal variables after covariate adjustment. The network findings are therefore descriptive and complementary; higher node strength for EA should not be interpreted as showing that EA is the causal driver of the system.

The findings should be considered in light of several limitations. First, the cross-sectional design does not establish temporal ordering or causality; reverse and bidirectional associations among EA, anxiety, PA, and AI dependence remain possible. Second, all measures were self-reported, and although Harman’s test and the ULMC analysis suggested that common method variance was not dominant, method bias cannot be fully excluded. Third, the AI dependence scale was adapted from an affective dependence measure originally designed for interpersonal relationships. Although we added expert content-validity evaluation, adolescent pretest/cognitive feedback, strong reliability and factorial evidence, and supplementary discriminant-validity evidence against short-form video addiction, these findings should still be regarded as preliminary psychometric support rather than a final validation of the construct. The dataset did not include general AI use, problematic AI use, problematic internet use, smartphone dependence, functional impairment, or behavioral logs; future studies should examine convergent validity, discriminant validity, criterion-related validity, test–retest reliability, and cross-sample stability in independent samples. Fourth, the proposed attachment and emotion-regulation interpretation was only indirectly examined because attachment style, loneliness, social support, emotion-regulation strategies, academic stress, sleep duration, parental monitoring, and AI-use motives were not measured. Fifth, although participants were recruited from multiple schools, the convenience sampling strategy limits generalizability. Longitudinal, multi-informant, and experimental or intervention studies are needed to clarify temporal processes and practical significance.

## 5 Conclusion

Among 1,589 Chinese adolescents, EA was associated with stronger AI dependence, and anxiety represented a small but significant statistical mediator of this association. PA moderated the EA-anxiety association, with weaker associations observed at higher PA levels. These results highlight anxiety and PA as meaningful correlates in adolescent AI dependence, while underscoring the need for longitudinal and intervention research before causal or preventive claims can be made.

## Data Availability

The raw data supporting the conclusions of this article will be made available by the authors, without undue reservation.
